# Scientific research and innovation response to the COVID-19 pandemic in Peru

**DOI:** 10.12688/f1000research.51400.2

**Published:** 2021-11-12

**Authors:** Jeel Moya-Salazar, Lucia Gomez-Saenz, Betsy Cañari, Hans Contreras-Pulache

**Affiliations:** 1 Faculties of Health Science, Universidad Norbert Wiener, Lima, Lima, 51, Peru; 2 Department of Pathology, Hospital Nacional Docente Madre Niño San Bartolomé, Lima, Lima, 51, Peru

**Keywords:** COVID-19, research, funding, SARS-CoV-2, Peru

## Abstract

**Background: **COVID-19 has shaken countries at all levels, putting public health at risk. Global efforts have allocated funding for the development of research for the development of vaccines, digital tools, epidemiologic, social, and economic studies. Although these efforts have been developed worldwide, not all countries have prioritized the same topics and may have a different impact on solving problems and containing the spread of COVID-19.

**Methods:** A descriptive study was conducted with secondary data of "Special Projects COVID-19” in order to analyze the prioritization of proposals and projects to Peruvian needs in the face of pandemic. Two calls were made by the Peruvian research council (CONCyTec); the first with five areas and the second with seven. The global amounts financed by each call were 342,857 USD (1,200,000 soles) and 700,000 USD (1,750,000 soles), respectively.

**Results: **A total of 1,101 research projects were presented, 600 (54.5%) in the first call. In this call, 176 (29.3%) projects were from technological development and innovation and 29 were winners (with a global budget of 1,711,907.25 USD /6,077,270.75 soles). In the second call, 120 (23.9%) projects were from the area of Social and economic research and 21 were winners (global budget of 1,284,002.25 USD/558,208.55 soles) (p=0.043). The largest proportion of winning projects in both calls was 12 (41.4%) in Technological developments and innovation, then five (17.2%) each in telehealth and mobile health, and epidemiological and social studies. Across both calls, 214 (55.8%) and 160 (51.9%) projects were of private organizations and universities, respectively.

**Conclusions: **This research shows ~2% of rapid response "Special Projects COVID-19” were financed by the CONCyTec call with over a million dollars of funds. Although the main topics were technological innovation, detection systems, and vaccines, these priorities have not had a global impact on the epidemiological development of the pandemic in Peru.

## Introduction

COVID-19 is an emerging infectious disease caused by the severe acute respiratory syndrome coronavirus 2 (SARS-CoV-2) belong to the coronaviridae family and is usually associated with mild-and-upper respiratory tract infections. SARS-CoV-2 is highly infectious, with an estimated mortality rate of between 2-4%, and high incidence of complications in the respiratory system (~29%), mainly in populations with risk factors and chronic diseases, (such as obesity, diabetes mellitus, hypertension, etc.). ^1^^,^^2^ This led almost all countries to opt for compulsory social isolation through quarantines, which led to the development and establishment of virtualization for educational, labor, and social purposes. ^3^^,^^4^

The COVID-19 crisis in Latin America afflicts communities with complex epidemiological profiles and social problems such as Venezuelan migration and corruption. ^5^ For these reasons, it is considered that the impact generated by this disease not only has repercussions on health but also is aggravating the economic and socio-political crises, forcing countries to take actions to reduce their progress even at the cost of their political-economic stability. ^5^ In their eagerness to face the pandemic, countries adopted preparedness and risk controls, where it established the creation of strategies that allow a correct and timely approach to patients, so they seek to provide diagnostic methods, mechanical ventilators, personal protective equipment (PPE), and health professionals. ^6^^,^^7^

All current technology is being deployed to face the health emergency due to COVID-19. Therefore governments, understanding the importance of science and technology, are providing funds for scientific and technological research and innovation to promote the development of rapid-response projects generally in infrastructure, equipment, diagnosis, and therapy of COVID-19. ^5^

In Peru, there is the National Council of Science, Technology and Technological Innovation (CONCyTec), which has the mission of formulating policies, and promoting and managing actions to create and disseminate knowledge and develop the social and economic aspects of the country, is providing financing facilities for innovative projects in the face of the COVID-19 pandemic “Special Projects” (CPSP) in the cultural, social, and health context. The impact of these government-funded projects should have a decisive impact on the course of the pandemic, providing solutions and generating knowledge as a means to understand, confront and control SARS-CoV-2, just as other countries are doing. ^8^^–^^11^

Since the strict restrictions due to the pandemic have caused far-reaching economic and social effects, mainly among the rural population, the CPSPs have been understood as solutions to the limitations imposed by COVID-19 in Peru. ^12^^–^^14^ Amidst a limited national research policy, ^15^ CONCyTEC has developed the calls thinking about solving the main problems related to the transmission dynamics of COVID-19 (i.e., mobility, rate of infection by region, report, follow-up of cases, and characteristics of SARS-CoV-2 deaths) and the socioeconomic consequences it leaves behind.

The objective of this research was to analyze the proposals and projects under the development of CPSP in the prioritization axis and as a proportional response to the needs in the face of COVID-19. Likewise, the prioritized thematic areas and the economic scope in the face of the COVID-19 pandemic in Peru were analyzed.

## Methods

### Design and data source

A descriptive cross-sectional study was designed with secondary data analysis of CPSP in the two calls launched by the government in search and recruitment of projects in science and technology during April - July 2020. The search parameters were all the projects presented and selected in both stages of the contest, following the admission and selection criteria of the CPSP: relevance, feasibility, and sustainability and impact of the proposals. There were no project limits in both calls. We searched the CPSP project database between November 10 and November 30, 2020. The underlying data used in this study is available on the CPSP site: https://fondecyt.gob.pe/convocatorias/innovacion-y-transferencia-tecnologica/proyectos-especiales-respuesta-al-covid-19.

### First call

Due to the lockdown by COVID-19, the Peruvian government through CONCyTec launched the first open call CPSP with an opening date of March 31, 2020, having two end dates: the first cutoff date, which is April 27, and the second cutoff date, which ended on April 30, 2020, to search for projects in five thematic areas assigned in the call:

1. Development and/or validation of detection systems
2. Telehealth and mobile health
3. Technological developments and innovation
4. Treatment
5. Epidemiological and social studies.

**Figure 1.**
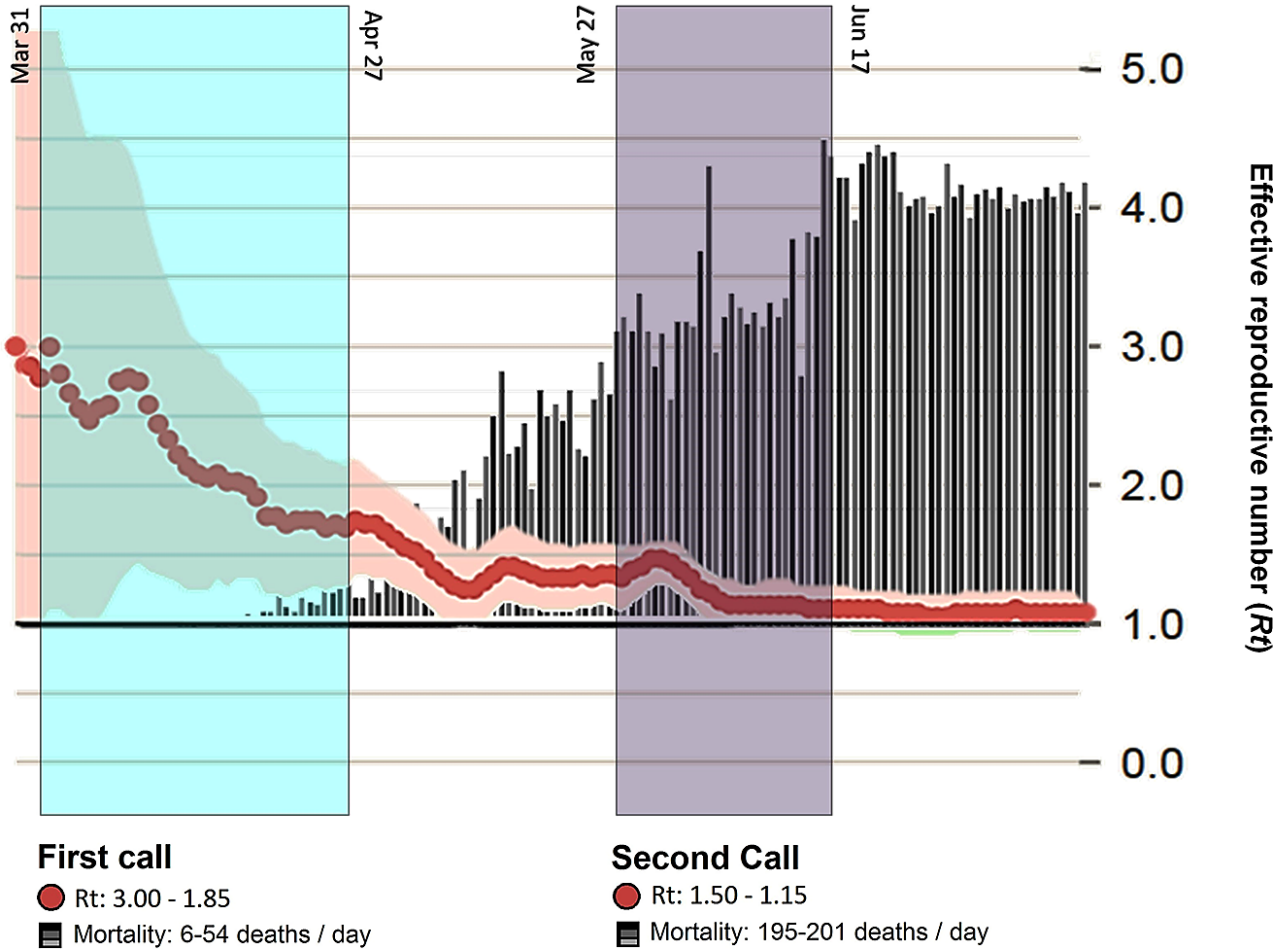
Peruvian research calls during the pandemic. Development of calls for research projects according to the trend line of cases (R, red line), mortality rate (black and white bars) and period of time of the study.

The call was divided into stages and had a maximum funding of 42,857.20 USD (150,000 soles), 80,000 USD (200,000 soles), 120,000 USD (300,000.00 soles), and 140,000 USD (350,000 soles) for each subject respectively, and 342,857 USD (1,200,000 soles) overall. 600 research projects were presented that included the participation of national and private universities, national institutes, and the private sector ( Figure 1). 51 projects were shortlisted in this call at the end of April according to CPSP criteria. From this list the winners were then selected. (Full list of project applicants and winners are available at: https://fondecyt.gob.pe/convocatorias/innovacion-y-transferencia-tecnologica/proyectos-especiales-respuesta-al-covid-19).

### Second call

Between May 27, and June 17, 2020, the second open call for “Special Projects: Modality-Emerging Needs to COVID-19 2020-02”. (Data available at: https://fondecyt.gob.pe/convocatorias/innovacion-y-transferencia-tecnologica/proyectos-especiales-modalidad-necesidades-emergentes-covid-19) This call-maintained distribution in seven thematic areas:

1. Treatment and transmission of SARS-Cov-2
2. Vaccines, antigens, and antivirals
3. Diagnosis
4. Surveillance and digital health
5. Sanitary devices
6. Epidemiology and prevention
7. Social and economic research

The call had a maximum funding of 85,714.28 USD (300,000 soles), 100,000 USD (350,000 soles), 85,714.28 USD (300,000.00 soles), 42,857.20 USD (150,000 soles), 57,142.85 USD (200,000 soles), 85,714.28 USD (300,000 soles), and 42,857.20 USD (150,000 soles), for each area, respectively. The total financing was 500,000 USD (1,750,000 soles) for all projects. The call followed the same contest criteria as the first, except that they had different general themes. As of July 11, 2020, 501 research projects were submitted, of which 125 were preselected according to CPSP criteria ( Figure 1).

### Measures of variables

From the websites of both calls, free access data was collected on the projects presented in the five and seven categories in the first and second calls, respectively. The variables included for the study were: General topic, requesting entity, and approved budget. The data was included and coded in a data matrix in MS-Excel 2013 v15.0 for Chrome (Google, CA, US), where it was subcategorized by the areas, calls, and project status as listed on the CPSP site. To avoid errors in data collection, two independent researchers participated in this process and two quality evaluations were carried out on the matrix obtained. As official data was used, no biases were identified.

### Data analysis procedure

The data analysis initially included a descriptive evaluation in both calls, presenting the findings of the category and continuous variables in frequencies. To show differences between calls and subject areas, we used non-paired Student's T-test and Tchi-Square considering a value of p <0.05 as significant. The statistical analyzer was SPSS v21.0 (IBM, Armonk, US) for Windows.

## Results

A total of 1,101 research projects were presented, 600 (54.5%) in the first call. For this call, 176 (29.3%) projects were from the technological development and innovation area, 164 (27.3%) from the telehealth and mobile health area, 95 (15.8%) were from the epidemiology and social studies area, 84 (14%) from the treatment area, and 81 (13.5%) in the area for development and/or validation of detection systems. In the second call, 501 projects were presented, of which 120 (23.9%) were from the area of social and economic research, 105 (20.9%) from epidemiology and prevention, 101 (20.1%) from sanitary accessories, 59 (11.7%) from surveillance and digital health, 54 (10.7%) from treatment and transmission of SARS-CoV-2, 47 (9.3%) from diagnosis, and 15 (2.9%) from vaccines, antigens, and antivirals ( Table 1).

**Table 1.** Total projects from both calls. Projects according to themes of the Special Projects: Response to COVID-19 contest. Data in n (%).

General Themes	Total Projects n (%)
First call	
Epidemiological and social studies	95 (15.8)
Development and/or validation of detection systems	81 (13.5)
Technological developments and innovation	176 (29.3)
Telehealth and mobile health	164 (27.3)
Treatment	84 (14)
Total	600 (100)
Second call	
Sanitary accessories	101 (20.1)
Diagnosis	47 (9.3)
Epidemiology and prevention	105 (20.9)
Social and economic research	120 (23.9)
Treatment and transmission of sars-cov-2	54 (10.7)
Vaccines, antigens and antivirals	15 (2.9)
Surveillance and digital health	59 (11.7)
Total	501 (100)

The organizations that participated in both calls are detailed in Table 2. In the first call, 214 (55.8%) organizations were private entities, 137 (35.7%) organizations were universities, 15 (3.9%) were public entities, and 17 (4.4%) were from the Ministry of Health of Peru (MINSA). In the second call, 51.9% (160 organizations) of organizations were universities, followed by 39.9% (123 organizations) private entities, 2.5% (eight organizations) public entities, and 5.5% (17 organizations) MINSA institutions. We found differences between the types (national, private, etc.) of participating institutions in both calls (p = 0.033).

**Table 2.** Projects according to organizations. Total of participating entities according to themes of the Special Projects: Response to COVID-19 contest for both calls. Data in n (%).

General Themes	Organizations				Total n (%)
	Universities n (%)	Private n (%)	Public n (%)	MINSA n (%)	
First call					
Development and/or Validation of Detection Systems	23 (46.9)	21 (42.9)	0 (0)	5 (10.2)	49 (12.8)
Treatment	21 (51.2)	18 (43.9)	2 (4.9)	1 (2.4)	41 (10.7)
Telehealth and Mobile Health	31 (26.7)	78 (67.2)	3 (2.6)	4 (3.4)	116 (30.3)
Technological Developments and Innovation	30 (25.2)	79 (66.4)	7 (5.9)	3 (2.5)	119 (31.1)
Epidemiological and Social Studies	33 (56.9)	18 (31)	3 (5.2)	4 (6.9)	58 (15.1)
Total	137 (35.7)	214 (55.8)	15 (3.9)	17 (4.4)	383 (100)
Second call					
Sanitary accessories	25 (43.1)	30 (51.7)	2 (3.4)	1 (1.7)	58 (18.8)
Diagnosis	19 (61.3)	10 (32.3)	0 (0)	2 (6.5)	31 (10.1)
Epidemiology and prevention	28 (45.2)	24 (38.7)	4 (6.5)	6 (3.2)	62 (20.1)
Social and economic research	35 (50)	32 (45.7)	1 (1.4)	2 (2.9)	70 (22.7)
Treatment and transmission of SARS-CoV-2	27 (75)	6 (16.7)	1 (2.8)	2 (5.6)	36 (11.9)
Vaccines, antigens, and antivirals	6 (75)	1 (12.5)	0 (0)	1 (12.5)	8 (2.6)
Surveillance and digital health	20 (46.5)	20 (46.5)	0 (0)	3 (7)	43 (14)
Total	160 (51.9)	123 (39.9)	8 (2.5)	17 (5.5)	308 (100)

**Figure 2.**
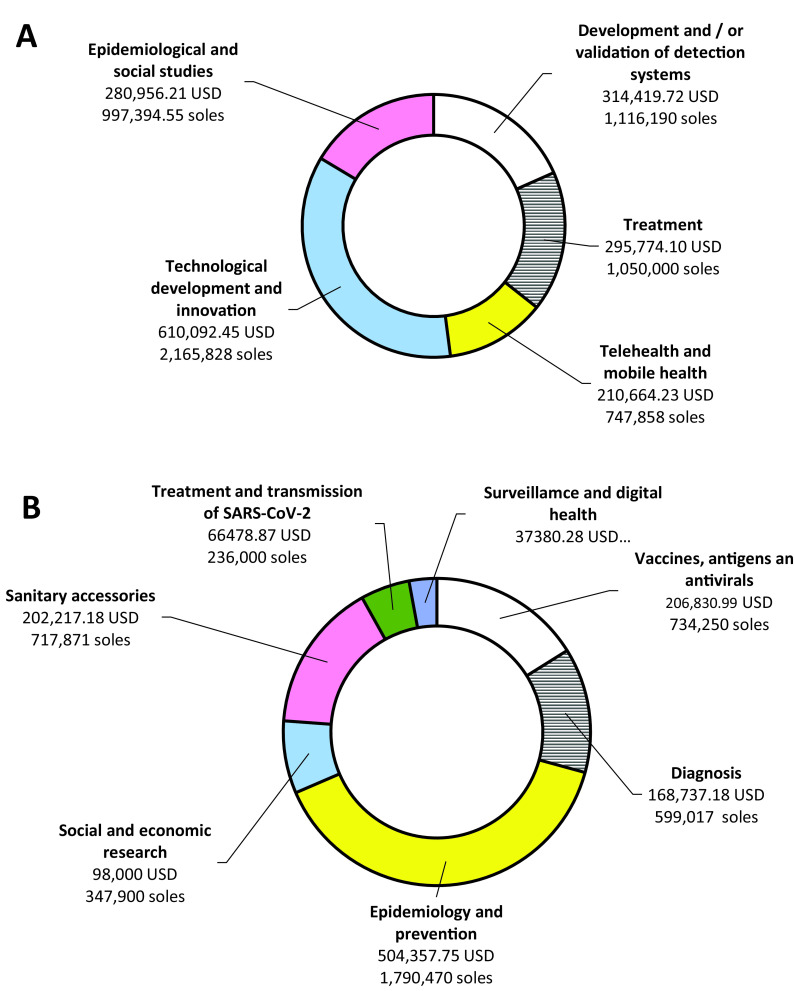
Projects financed in the two calls. Budget approved for each theme for the winning projects of the Special Projects: Response to COVID-19 contest for first (A) and second (B) calls.

Total winning projects for both calls was 50 (8.3%) projects with a global budget of 2,995,909.51 USD (10,635,478.75 soles). According to the selection of projects, 29 winners were obtained in the first call, with 12 (41.4%) in technological developments and innovation, and five (17.2%) in each telehealth and mobile health, and epidemiological and social studies ( Figure 2). For these projects, a global budget of ~1.7 million dollars (6,077,270.75 soles) was distributed as 35.6% (610,092.45 USD/2,165,828.20 soles) allocated for projects in technological development and innovation, and 16.4% (280,956.21 USD/997,394.55 soles) for projects to epidemiological and social. For the second call, there were a total of 21 winners, six (28.6%) in Epidemiology and prevention and five (23.8%) in Sanitary accessories, with a global assigned budget of 1,284,002.25 USD (4,558,208.55 soles) ( Table 3). We found differences in funding amounts between calls (p = 0.043) and between the areas within each call (p = 0.010).

**Table 3.** Financing distribution according to thematic in both calls.

Calls	Thematic area			Financing	
				USD	Soles
First call	Technological development and innovation (N = 12)			610,092.45	2,165,828.00
	Development and/or validation of detection systems (N = 4)			314,419.72	1,116,190.00
	Treatment (N = 3)			295,774.10	1,050,000.00
	Epidemiological and social studies (N = 5)			280,956.21	997,394.55
	Telehealth and mobile health (N = 5)			210,664.23	747,858.00
	**Total**			1,711,907.25	6,077,270.75
Second call	Sanitary accessories (N = 5)			202,217.18	717,871.00
	Diagnosis (N = 2)			168,737.18	599,017.00
	Epidemiology and prevention (N = 6)			504,357.75	1,790,470.00
	Social and economic research (N = 3)			98,000.00	347,900.00
	Treatment and transmission of SARS-CoV-2 (N = 1)			66,478.87	236,000.00
	Vaccines, antigens and antivirals (N = 3)			206,830.99	734,250.00
	Surveillance and digital health (N = 1)			37,380.28	132,700.00
	**Total**			1,284,002.25	4,558,208.55

The main universities responsible for the winning projects were Universidad Peruana Cayetano Heredia (UPCH) (n = 10, 20%), the Pontifica Universidad Católica del Perú (PUCP) (n = 7, 14%), and the Universidad Nacional Mayor de San Marcos (n = 3, 6%). Other institutions with winning projects were the National Health Institute of Peru (n = 4, 8%) and Farmacológicos Veterinarios S.A.C. (n = 2, 4%) corresponding to the Ministry of Health and the private sector, respectively.

## Discussion

In this study, we determined that Peru has allocated more than 2.9 million dollars for research on COVID-19 in order to obtain the necessary tools to face the inclemency of the pandemic. Of the 50 award-winning projects, those prioritized were those of technological development and innovation, telehealth and mobile health, and epidemiological and social studies, proposed mainly by private institutions.

Although Peruvian institutions have shown interest in promoting the efforts to meet the needs of the COVID-19 pandemic and the extensive measures to reduce contagion and mortality rates, the results have shown the opposite. Peru has been the first country to foresee prevention activities against COVID-19, these strategies have shown promise and have involved high costs due to prolonged quarantine, social immobility, and SARS-CoV-2 detection tests. ^5^ However, during the last six months it has become the opposite. Peru’s double problem is that it is the first country with the largest number of deaths per 100,000 inhabitants and, due to its political complexity, its potential future economic recession (GDP -15.5%) is the largest in the United States. ^16^^,^^17^

This situation also seems to affect Peruvian science and technology. Efforts to promote technological innovation and research projects in the sciences and humanities are being intransigent, due to the low impact they have achieved. The onsets of the pandemic brought concern about the supply of diagnostic tests throughout the world, and, in Peru with funding from CONCyTec, funds were directed to the development of molecular detection and diagnostic tests (and also in-house systems of oxygen supply). Contrary to expectations, more than six projects ^18^ are still waiting to complete the validation and approval stages of the government patent bodies (Instituto Nacional de Defensa de la Competencia y de la Protección de la Propiedad Intelectual - INDECOPI) that are not adequately planned and are being “built” together with the progress of the pandemic.

Although Peru does not have an industry specifically designed for mass production of vaccines or diagnostic tests, the government has prioritized the development of these projects ( Tables 2 and 3). Most of the government funds for R+D+i have been directed to the development of Peruvian vaccines against SARS-CoV-2. ^19^ Also, in this context, the most formulated public policies have impeded their advance, despite their inclusion on the list of vaccine candidates by the World Health Organization. ^20^

In Peru, CONCYTEC presented two calls, accepted 50 projects, and provided USD 2,995,909.51 (10,635,478.75 soles) in financing ( Table 3). The purposes of these projects have been based on the health realities of other countries with high rates of mortality and contagion, although they differ through the San Carlos III Institute with a budget of € 24 million with the goal of financing projects that increase knowledge of the management of the disease to optimize the response to the pandemic. ^10^ This initiative has financed a total of 132 projects, prioritizing the proposals for rapid diagnosis techniques of the virus, biologic-molecular clinic, prognosis, complications of COVID-19, vaccine development, epidemiological surveillance, socio-economic impact and artificial intelligence, and the massive analysis of integrated data oriented to epidemiological control. On the other hand, the Spanish BBVA Foundation has funded research on biomedicine (Biomed-COVID-19), big data and artificial intelligence (Data-IA-COVID-19), ecology and veterinary medicine (Eco-Vet-COVID-19), economics and social sciences (Socioecon-COVID-19), humanities (Human-COVID-19) with € 250,000, € 150,000, € 100,000, € 100,000, and € 75,000, respectively. ^21^

In Latin America, several countries have intensified their support and interest in research and scientific innovation on COVID-19, such as the calls from Argentina (PISAC-COVID 19), Mexico (CONACYT), Chile (ANID), Costa Rica (UCREA), and Ecuador (United States Embassy - Opportunity Funds for the year 2020). Also, the National Agency for Research and Development of Chile made a call that embraced the diagnosis, treatment, prevention, control, or aspects from the technological, social, cultural, economic, and humanistic field of COVID-19, whereof a total of 1,055 projects, 63 (5.9%) were approved and the budget for each project was 11,608.41 USD (731,304 USD in total). ^9^^,^^22^^,^^23^

In the case of the United States, the National Institutes of Health (NIH) and Johns Hopkins University have made international efforts to develop scientific and technological projects. Until August 2020, the NIH approved a total of 653 projects, one of them with the largest budget of all the projects analyzed: characterizing SARS-COV-2-Specific Immunity in Convalescent Individual with a total of 138,462 987 USD. Johns Hopkins University granted funding for August 2020, to a total of 77 projects, where two projects reached the highest budget with USD 22 million for both: Phased Large Awards in Comparative Effectiveness Research (PLACER) and NEW Making a Difference Request for Proposals - Fall 2020. This university has also been developing global technological development events, such as the Johns Hopkins Healthcare Design Competition 2020 and MedHacks 2020, with budgets of more than 30,000 USD. ^8^

In Peru, there are no direct political regulations on scientific production that allow the guidance of scientific and technological projects that benefit society. ^15^ In fact, during the COVID-19 pandemic, Peru has reported one of the lowest scientific productions in Latin America and the Caribbean. ^24^ In this context, the CPSPs must improve these indicators since they have high government investment. It is also necessary to know the regulation of these projects and how they have impacted the community, if they have limited personal financial interests and, if they have promoted a better response to COVID-19 since many countries with large populations have presented containment weaknesses. ^25^^-^^28^

This study had limitations: 1. We did not have access to the comprehensive review of the winning projects, to know their objectives and deadlines; 2. We were unable to follow up on the projects, to find out how many projects met their objectives and how many gave up. Despite these limitations, this study presents for the first time the analysis of rapid response projects against COVID-19 in Peru.

## Conclusions

In conclusion, in Peru around 8% of CPSP against COVID-19 were financed after being submitted to the CONCyTec call with more than 2.9 million US dollars of funds allocated. Although the main topics were technological innovation, mobile health, and epidemiological and social studies, these priorities have not had an impact on the epidemiologic development of the pandemic in the country. It is necessary to improve public policies that limit the rapid application of these projects as part of the strategies to face the pandemic, and the social and economic consequences that it is leaving. These results can help political leaders responsible for Peruvian science and technology to formulate better policy guidelines, redesign the financial-scientific-strategic mosaic and link social agents to infer research priorities to improve the response to COVID -19 and to face future epidemic threats.

## Data Availability

All data underlying the results are available as part of the article and no additional source data are required.
