# Supplementary material for: Scientific research and innovation response to the COVID-19 pandemic in Peru
Source: F1000Res. 2021 Nov 12;10:399. Originally published 2021 May 18. [Version 2] doi: 10.12688/f1000research.51400.2 (PMC8609394; doi:10.12688/f1000research.51400.2)
Supplement: Peer review history

# Peer review: Reviewer response for version 2

**DOI:** 10.5256/f1000research.79217.r100048

**Published:** 2021-11-22

**Authors:** Mario Coccia

**Affiliations:** 1 CNR, National Research Council of Italy, Moncalieri, Italy

I have read thoroughly the revised version of paper.

 The authors have done considerable additional work, and addressed all concerns and criticisms in the revised manuscript, which I believe has improved substantially in the theoretical framework, study design and discussion of results.

 Now, the paper is OK.

Is the work clearly and accurately presented and does it cite the current literature?

No

If applicable, is the statistical analysis and its interpretation appropriate?

Partly

Are all the source data underlying the results available to ensure full reproducibility?

Partly

Is the study design appropriate and is the work technically sound?

Partly

Are the conclusions drawn adequately supported by the results?

No

Are sufficient details of methods and analysis provided to allow replication by others?

Partly

Reviewer Expertise:

COVID-19 studies

I confirm that I have read this submission and believe that I have an appropriate level of expertise to confirm that it is of an acceptable scientific standard.

# Peer review: Reviewer response for version 1

**DOI:** 10.5256/f1000research.54563.r94812

**Published:** 2021-10-01

**Authors:** Mario Coccia

**Affiliations:** 1 CNR, National Research Council of Italy, Moncalieri, Italy

Scientific research and innovation response to the COVID-19 pandemic in Peru

 The topics of this paper are interesting but the structure and content have to be revised by authors before being considered for indexing.

1. The abstract needs to show briefly, health, research, and social policy implications. 
2. The introduction needs to better clarify the research questions of this study. Moreover, authors need to better describe the different sources of transmission dynamics of COVID-19 (e.g., mobility, environment, etc.) and risk factors in society, and restriction policies that affect the socioeconomic system of Peru (see suggested readings that have to be all read and used in the text). 
3. The methods of this study are not clear. The section of Materials and methods needs to be restructured with the following three sections: Sample and data, and research settings,  Measures of variables, and data analysis procedure. 
4. Results. Figure 1 needs to show the measure of the variable on the y-axis. The legend has to be in English.
5. Discussion can show, if possible, the implications of these calls in terms of scientific output (publications or patents) to assess the effects on basic and applied research. Suggested papers, including some of mine, are important to reinforce the theoretical framework and support a better discussion and best practices of the scientific and technological change to face the COVID-19 pandemic crisis. 
6. The conclusion has not to be a summary but authors have to focus on manifold limitations of this study and provide suggestions of health and social policy and strategies of prevention.

Overall the paper is interesting but the theoretical framework is weak, and some results create confusion… structure of the paper has to be improved; study design, discussion, and presentation of results have to be clarified using suggested comments.

 If the paper is improved,  by using all comments,  maybe it can be considered.

 Suggested readings of relevant and new papers that have to be read and all inserted in the text and references.

 Alves, I., Analide, C., Vaz, F.   2022. The Relationship Between Financial Execution in R&D and Scientific Production  Lecture Notes in Networks and Systems, 332, pp. 171-181 ^1^

 Coccia M. 2022. Preparedness of countries to face covid-19 pandemic crisis: Strategic positioning and underlying structural factors to support strategies of prevention of pandemic threats, Environmental Research, n. 111678 ^2^.

 Durán, R.L.2021. COVID-19 and heterogeneous vulnerabilities in the Peruvian labor market: implications for social inequalities and for gender gaps,  Economia Politica ^3^

 Coccia M. 2021. Effects of the spread of COVID-19 on public health of polluted cities: results of the first wave for explaining the dejà vu in the second wave of COVID-19 pandemic and epidemics of future vital agents. Environmental Science and Pollution Research. ^4^

 Portocarrero Merino, E., Veramendi Villavicencios, N.G., Enrique Suero Rojas, E., Mendoza Loli, J.S. 2021. Development and validation of scale resilient coverage and future expectations in university of huanuco in times of COVID-19 | [Desarrollo y validación de escala afrontamiento resiliente y expectativas de futuro en universitarios de huanuco en tiempos de COVID-19], Universidad y Sociedad, 13(4), pp. 132-142 ^5^

 Coccia M. 2021. Pandemic Prevention: Lessons from COVID-19. Encyclopedia 2021, 1, 433–444. MDPI, Basel, Switzerland, Encyclopedia of COVID-19 ISSN 2673-8392,  journal, (https://www.mdpi.com/journal/encyclopedia) ^6^

 Kinnunen, J., Georgescu, I., Hosseini, Z., Androniceanu, A.-M.    2021, Dynamic indexing and clustering of government strategies to mitigate Covid-19,     Entrepreneurial Business and Economics Review, 9(2), pp. 7-20 ^7^

 Coccia M. 2021. High health expenditures and low exposure of population to air pollution as critical factors that can reduce fatality rate in COVID-19 pandemic crisis: a global analysis. Environmental Research, vol. 199, Article number 111339, ^8^

 Barreto, I.B., Sánchez, R.M.S., Marchan, H.A.S. 2021, Economic and social consequences of human immobility due to COVID-19: Peru case study | [Consecuencias económicas y sociales de la inamovilidad humana bajo Covid – 19 caso de estudio Perú],     Lecturas de Economia, (94), pp. 285-303 ^9^

 Tang, X., Wu, C., Li, X., Song, Y., Yao, X., Wu, X., Duan, Y., (...), Lu, J. 2020. On the origin and continuing evolution of SARS-CoV-2 , (2020) National Science Review, 7 (6), pp. 1012-1023. ^10^

Is the work clearly and accurately presented and does it cite the current literature?

No

If applicable, is the statistical analysis and its interpretation appropriate?

Partly

Are all the source data underlying the results available to ensure full reproducibility?

Partly

Is the study design appropriate and is the work technically sound?

Partly

Are the conclusions drawn adequately supported by the results?

No

Are sufficient details of methods and analysis provided to allow replication by others?

Partly

Reviewer Expertise:

COVID-19 studies

I confirm that I have read this submission and believe that I have an appropriate level of expertise to confirm that it is of an acceptable scientific standard, however I have significant reservations, as outlined above.

## References

[1] : The Relationship Between Financial Execution in Research and Development and Scientific Production. *Springer Cham* .2022;332: 10.1007/978-3-030-86887-1_16 10.1007/978-3-030-86887-1_16

[2] : Preparedness of countries to face covid-19 pandemic crisis: Strategic positioning and underlying structural factors to support strategies of prevention of pandemic threats. *Environ Res* .2021; 10.1016/j.envres.2021.111678 111678 10.1016/j.envres.2021.111678 34280421PMC8284056

[3] : COVID-19 and heterogeneous vulnerabilities in the Peruvian labor market: implications for social inequalities and for gender gaps. *Economia Politica* .2021; 10.1007/s40888-021-00245-5 10.1007/s40888-021-00245-5 PMC845970335422578

[4] : Development and validation of scale resilient coverage and future expectations in university of huanuco in times of COVID-19. *Revista Universidad Y Sociedad* .132-142

[5] : Effects of the spread of COVID-19 on public health of polluted cities: results of the first wave for explaining the dejà vu in the second wave of COVID-19 pandemic and epidemics of future vital agents. *Environ Sci Pollut Res Int* .2021;28(15) : 10.1007/s11356-020-11662-7 19147-19154 10.1007/s11356-020-11662-7 33398753PMC7781409

[6] : Pandemic Prevention: Lessons from COVID-19. *Encyclopedia* .2021;1(2) : 10.3390/encyclopedia1020036 433-444 10.3390/encyclopedia1020036

[7] : Dynamic indexing and clustering of government strategies to mitigate Covid-19. *Entrepreneurial Business and Economics Review* .2021;9(2) : 10.15678/EBER.2021.090201 7-20 10.15678/EBER.2021.090201

[8] : High health expenditures and low exposure of population to air pollution as critical factors that can reduce fatality rate in COVID-19 pandemic crisis: a global analysis. *Environ Res* .199: 10.1016/j.envres.2021.111339 111339 10.1016/j.envres.2021.111339 34029545PMC8139437

[9] : Consecuencias económicas y sociales de la inamovilidad humana bajo Covid – 19 caso de estudio Perú. *Lecturas de Economía* .2021; 10.17533/udea.le.n94a344397 285-303 10.17533/udea.le.n94a344397

[10] : On the origin and continuing evolution of SARS-CoV-2. *National Science Review* .2020;7(6) : 10.1093/nsr/nwaa036 1012-1023 10.1093/nsr/nwaa036 PMC710787534676127

# Response

**Published:** 2021-11-06

**Authors:** Jeel Moya-Salazar

**Affiliations:** Universidad Norbert Wiener, Peru

Dear Dr. Coccia, 

 We have received and reviewed your comments, and we welcome your suggestions so that we can improve our research. All changes have been made in the abstract, introduction, methodology, discussion, and conclusions as indicated.

 We have reviewed the studies you suggested and included some of them as part of the research. Figure 1 that had some Spanish terms has been adjusted.

 We hope this new version of the study in Peru will improve your background in research and development. We have learned a lot from the research we have read during the corrections.

 Sincerely,

# Peer review: Reviewer response for version 1

**DOI:** 10.5256/f1000research.54563.r85661

**Published:** 2021-07-23

**Authors:** Rodrigo Ernesto Salazar Gamarra

**Affiliations:** 1 Paulista University (UNIP), Sao Paulo, Brazil

From a public health perspective, it is important to understand how the priorities of government instances and stakeholders work together to allow the community to work for its society. In that sense, the objective of the present research has a high-importance relevance to analyze this situation for the Peruvian population. 

 This research touches on funding assignments for the scientific community called CONCYTEC. This is the principal grant opportunity for Peruvian scientists to execute their projects and it is important to do a discussion around how it impacted this pandemic time. Results showed that there is still important work around public policies improvement.

 I agree with the indexing of this article.

Is the work clearly and accurately presented and does it cite the current literature?

Yes

If applicable, is the statistical analysis and its interpretation appropriate?

Yes

Are all the source data underlying the results available to ensure full reproducibility?

Yes

Is the study design appropriate and is the work technically sound?

Yes

Are the conclusions drawn adequately supported by the results?

Yes

Are sufficient details of methods and analysis provided to allow replication by others?

Yes

Reviewer Expertise:

Public healthcare & Healthcare innovation

I confirm that I have read this submission and believe that I have an appropriate level of expertise to confirm that it is of an acceptable scientific standard.
